# The functional significance of microRNA-145 in prostate cancer

**DOI:** 10.1038/sj.bjc.6605742

**Published:** 2010-06-29

**Authors:** M S Zaman, Y Chen, G Deng, V Shahryari, S O Suh, S Saini, S Majid, J Liu, G Khatri, Y Tanaka, R Dahiya

**Affiliations:** 1 Department of Urology, San Francisco Veterans Affairs Medical Center and University of California at San Francisco, San Francisco, CA, USA

**Keywords:** miR145, prostate, cell growth, apoptosis, TNFSF10

## Abstract

**Background:**

MicroRNAs (miRNAs) are small noncoding RNAs that have important roles in numerous cellular processes. Recent studies have shown aberrant expression of miRNAs in prostate cancer tissues and cell lines. On the basis of miRNA microarray data, we found that miR-145 is significantly downregulated in prostate cancer.

**Methods and results:**

We investigated the expression and functional significance of miR-145 in prostate cancer. The expression of miR-145 was low in all the prostate cell lines tested (PC3, LNCaP and DU145) compared with the normal cell line, PWR-1E, and in cancerous regions of human prostate tissue when compared with the matched adjacent normal. Overexpression of miR-145 in PC3-transfected cells resulted in increased apoptosis and an increase in cells in the G2/M phase, as detected by flow cytometry. Investigation of the mechanisms of inactivation of miR-145 through epigenetic pathways revealed significant DNA methylation of the miR-145 promoter region in prostate cancer cell lines. Microarray analyses of miR-145-overexpressing PC3 cells showed upregulation of the pro-apoptotic gene *TNFSF10*, which was confirmed by real-time PCR and western analysis.

**Conclusion:**

One of the genes significantly upregulated by miR-145 overexpression is the proapoptotic gene *TNFSF10*. Therefore, modulation of miR-145 may be an important therapeutic approach for the management of prostate cancer.

Micro RNAs (miRNAs) are small noncoding RNAs that regulate the expression of protein-coding genes (He and Hannon, 2004; Lim *et al*, 2005; Volinia *et al*, 2006). MicroRNAs have important roles in numerous cellular processes, including development, proliferation and apoptosis (Carrington and Ambros, 2003). Characteristic miRNA signatures for several epithelial cancers, including breast, lung, pancreatic and gastric cancers, have been reported (Iorio *et al*, 2005; Yanaihara *et al*, 2006; Bloomston *et al*, 2007; Petrocca *et al*, 2008). Micro RNAs control gene expression by binding to complementary sites in the 3′ untranslated regions (3′ UTRs) of target mRNAs, triggering either translational inhibition or mRNA degradation (Baek *et al*, 2008). However, miRNAs can also function to positively regulate gene expression by binding to complementary or partial complementary sequences in the promoter regions of genes (Place *et al*, 2008). Recent studies have shown the aberrant expression of miRNAs in prostate cancer tissues and cell lines (Galardi *et al*, 2007; Porkka *et al*, 2007; Ozen *et al*, 2008). One of the miRNAs reported to be consistently downregulated in prostate tumours is miR-145, and it has been shown to act as a tumour suppressor in breast and colon cancers (Schepeler *et al*, 2008; Spizzo *et al*, 2009).

In this study we analysed the functional significance of miR-145 in prostate cancer. We examined the expression level of miR-145 in 27 matched human prostate cancer laser microdissected tissue samples, and found a significant difference between adjacent normal and cancerous regions. We also investigated whether the mechanism of inactivation of miR-145 in prostate cancer cell lines is through methylation of its promoter. After combination treatments with low doses of genistein (G), 5-aza-2′-deoxycytidine (5-aza) and trichostatin A (TSA), there was an increase in the expression of miR-145, suggesting that silencing of miR-145 is through DNA methylation. To identify potential miR-145 targets, we performed microarray analyses in miR-145-overexpressing prostate cancer cells and found upregulation of the pro-apoptotic gene *TNFSF10*. We also observed that overexpression of miR-145 in PC3 cells led to cell cycle arrest and increased apoptosis. This is the first report on the mechanisms of inactivation of miR-145 through DNA methylation in prostate cancer.

## Materials and methods

### Cell lines and cell culture

Human prostate carcinoma cell lines, PC3, LNCaP and Du145, and the normal epithelial prostate cell line, PWR-1E, were obtained from the American Type Culture Collection (ATCC, Manassas, VA, USA). The prostate cancer cell lines were cultured as monolayers in RPMI-1640 (UCSF Cell Culture Facility, San Francisco, CA, USA) supplemented with 10% foetal bovine serum (Hyclone, Logan, UT, USA), 50 mg ml^–1^ penicillin and 50 mg ml^–1^ streptomycin (Invitrogen, Carlsbad, CA, USA), and maintained in an incubator with a humidified atmosphere of 95% air and 5% CO_2_ at 37 °C. The PWR-1E cells were cultured in keratinocyte growth medium supplemented with 5 ng ml^–1^ human recombinant epidermal growth factor and 0.05 mg ml^–1^ bovine pituitary extract (Life Technologies/Invitrogen, Carlsbad, CA, USA) and maintained in an incubator under the conditions described above. Subconfluent cells (60–70% confluent) were treated with varying concentrations of genistein (25 *μ*M), 5-aza (5 *μ*M) and TSA (100 ng *μ*l^–1^). Genistein (Sigma-Aldrich Corp., St Louis, MO, USA) was dissolved in DMSO, and cells treated only with vehicle (DMSO) served as control. Fresh genistein and 5-aza (Sigma) were administered everyday along with a change of medium, and the cells were grown for 96 h. Trichostatin A (Upstate Biotech, Lake Placid, NY, USA) was administered for the last 24 h of treatment.

### Quantitative real-time PCR

First-strand cDNA was prepared from total RNA (1 *μ*g) using the reverse transcription system (Promega, Madison, WI, USA). Total RNA was extracted using the RNeasy mini kit from Qiagen (Valencia, CA, USA). In the real-time polymerase chain reaction (PCR) step, complementary DNA was amplified with Inventoried Gene Assay Products containing two gene-specific primers and one TaqMan MGB probe (6-FAM dye-labeled) using the TaqMan Universal Fast PCR Master Mix in a 7500 Fast Real-Time PCR System (Applied Biosystems, Foster City, CA, USA). Thermal cycling conditions included 95 °C for 20 s, 40 cycles of 95 °C for 3 s and 60 °C for 30 s according to the TaqMan Fast Universal PCR protocol. GAPDH was used as an endogenous control. For miRNA real-time experiments the cDNA strand was synthesised using Applied Biosystems Taqman MicroRNA Reverse Transcription kit, using a total of 200 ng of total extracted miRNA. RNU48 was used as an endogenous control. In real-time experiments, in which miRNAs were isolated from formalin-fixed paraffin-embedded (FFPE) prostate samples, let7-f was used as an endogenous control. Total miRNA was extracted from the FFPE samples using the miRNA FFPE kit from Qiagen.

### Laser capture microdissection (LCM) and RNA extraction from FFPE human prostate tumour samples

Adjacent normal and cancerous prostate tissues were obtained from 27 representative FFPE tissue blocks of radical prostatectomy specimens from the Pathology Department of the Veterans Affairs Medical Center of San Francisco. For the identification of prostatic adenocarcinoma as well as normal and hyperplastic glandular epithelium and stroma, the slides were reviewed by a certified pathologist. Microdissection of cancerous and adjacent normal regions was performed using the Arcturus Autopix system from Molecular Devices (Sunnyvale, CA, USA). In short, 8*μ*m sections were placed on glass slides, deparaffinised, stained with haematoxylin, dehydrated and placed in the Arcturus instrument for microdissection. Tissue samples of the different cancerous and adjacent normal regions were captured with infrared laser pulses onto CapSure Macro LCM caps and RNA was extracted following the instructions in the miRNA FFPE kit from Qiagen. The levels of miR-145 were assessed by the Taqman miR assay as described above. The relative miR-145 expression levels in cancerous regions were normalised to their adjacent normals (value 1).

### Sodium bisulphite modification and sequencing

Bisulphite modification of DNA was performed using the Epi-Tect Bisulphite kit (Qiagen) following the manufacturer's directions. The basic principle of bisulphite modification of DNA is that in the bisulphite reaction, all unmethylated cytosines are deaminated and sulphonated, converting them to thymines, whereas methylated cytonies (5-methylcytosines) remain unaltered. Thus, the sequence of the treated DNA will differ depending on whether the DNA is originally methylated or unmethylated. Primers for bisulphite genomic sequencing PCR were designed using the online program MethPrimer (Li and Dahiya, 2002). All reactions for tissue samples were subjected to two rounds of amplifications using a nested primer approach. Bisulphite-modified DNA (1 *μ*l) was amplified using a primer pair in a total volume of 20 *μ*l. Aliquots (2 *μ*l) of the first PCR reactions were subjected to second-round amplifications using a pair of nested primers in a total volume of 30 *μ*l. The amplification products were confirmed by electrophoresis on a 2% agarose gel and sequenced directly by an outside vendor (McLab, South San Francisco, CA, USA).

### Cell transfection

PC3 cells were transiently transfected with either precursors of miRNA-145 or negative control 1 (both from Ambion, Austin, TX, USA), using Lipofectamine reagent (Invitrogen), according to the manufacturer's recommendations. In brief, cells were seeded in 100 mm dishes (Nunc, Roskilde, Denmark) 24 h before transfection and were transiently transfected at a confluency of 40–50%. Mock transfection, which only had the transfection reagent, was also used as a control. The transfection mixture was dissolved in Opti-MEM serum-free media (Invitrogen) and at the time of transfection cells were seeded in RPMI-1640 media (UCSF Cell culture facility), with 10% FBS (Atlanta Biologicals, Lawrenceville, GA, USA) and no antibiotics. On the subsequent day the media was changed and RPMI media containing both FBS and 1% antibiotic (penicillin–streptomycin, 100 × , UCSF Cell Culture Facility) was added to the cells. Cells were pelleted after 72 h of transfection for flow cytometry, RNA and protein extraction.

### Cell proliferation assay

For cell proliferation assay, PC3 cells were seeded in 96-well microplates at a density of 5 × 10^3^ cells per well 24 h before transfection. After transfection, cell viability was determined at 24, 48 and 72 h using the CellTiter 96 Aqueous One Solution Cell Proliferation Assay kit (Promega) according to the manufacturer's protocol. Absorbance at 490 nm was measured with a kinetic microplate reader (Spectra MAX 190; Molecular Devices) and was used as a measure of cell number. Experiments were performed in quadruplicate and repeated three times.

### Cell cycle analysis

Cell cycle analysis was performed 72 h after transfection. The cells were harvested, washed with cold PBS, UCSF Cell Culture Facility, and resuspended in the nuclear stain 4′,6-diamidino-2-phenylindole (Beckman Coulter, Brea, CA, USA). Stained cells were immediately analysed with a flow cytometer (Cell Lab Quanta SC; Beckman Coulter).

### Apoptosis assay

For apoptosis, transfected cells at 72 h after transfection were dual stained with the viability dye 7-amino-actinomycin D and Annexin V-FITC using Annexin V-FITC/7-amino-actinomycin D kit (Beckman Coulter) according to the manufacturer's protocol. Stained cells were immediately analysed by flow cytometry (Cell Lab Quanta SC; Beckman Coulter).

### Western analysis

Whole-cell extracts were prepared in radioimmunoprecipitation assay buffer (RIPA; Thermo Scientific, Rockford, IL, USA; 50 mmol l^–1^ Tris (pH 8.0), 150 mmol l^–1^ NaCl, 0.5% deoxycholate, 0.1% SDS and 1.0% NP-40) containing 1 × protease inhibitor cocktail (Roche, Basel, Switzerland). Protein estimations were performed using a BCA Protein assay kit (Pierce/Thermo Scientific, Rockford, IL, USA) according to the manufacturer's instructions. Total protein (40 *μ*g) was electrophoresed in 15% SDS–PAGE gels, and western blotting was carried out using standard protocols and detected by chemiluminescence. All antibodies, which included TNFSF10 (cat. no. 3219), cdk6 (cat. no. 3136) and GAPDH (cat. no. 2118), were purchased from Cell Signaling (Danvers, MA, USA).

### Statistical analysis

Statistical analysis was performed using StatView version 5.0 for Windows as needed. Student's *t-*test was used to compare the different groups. The *P-*values of <0.05 were regarded as statistically significant.

## Results

### Expression of miR-145 in prostate cell lines and human tissues

**Figure 1:**
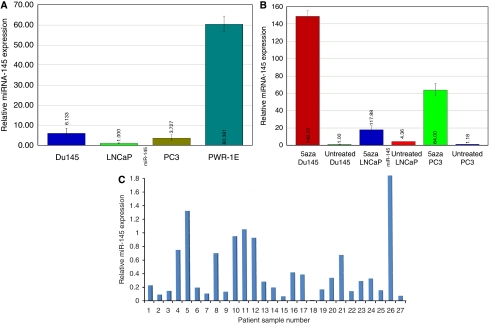
miR-145 expression is silenced in prostate cancer cell lines and tumour samples. (**A**) Level of miR-145 in untreated PC3, LNCaP, Du145 and PWR-1E cell lines. The expression of miR-145 was found to be low in all the prostate cancer cell lines tested (untreated PC3, LNCaP and Du145) when compared with the normal PWR-1E cells. (**B**) 5-aza treatment of prostate cancer cell lines resulted in a significant increase in the expression of miR-145. (**C**) Relative miR-145 levels in cancerous regions of prostate tissue, as normalised to adjacent normal (value 1). The level of miR-145 expression in adjacent normal prostate tissue regions was found to be significantly higher when compared with the matched cancerous regions in 80% of the samples tested.

We first checked the expression levels of miR-145 in prostate cell lines and human tissue samples. The cell lines used were PC3, LNCaP, Du145 and PWR-1E, in which PC3, LNCaP and Du145 were prostate cancer cell lines, whereas PWR-1E was a normal prostatic epithelial cell line. The expression of miR-145 was found to be low in all the prostate cancer cell lines tested (untreated PC3, LNCaP and Du145) when compared with the normal PWR-1E cells (Figure 1A). Treatment with 5-aza resulted in a significant increase in the expression of miR-145 in all cell lines, which suggests epigenetic silencing of this miRNA in the prostate cancer cell lines (Figure 1B). Subsequently, we examined 27 matched prostate cancer laser microdissected human tissue samples. The level of miR-145 expression in adjacent normal regions was found to be significantly higher when compared with matched cancerous regions in 80% of the samples tested (Figure 1C).

### miR-145 promoter CpG island methylation and increased expression with 5-aza treatment

**Figure 2:**
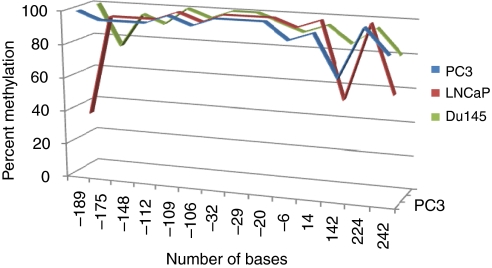
Methylation status of the 5′ upstream region of the miR-145 transcription start site in prostate cancer cell lines. The *x* axis denotes the number of bases. The +ve and −ve values attached to the bases represent downstream (+ve) and upstream (−ve) of the putative transcriptional start site of the precursor of miR-145. The *y* axis indicates the percentage of methylation in the CpG sites.

To elucidate the low expression of miR-145 in prostate cells and tumours, we analysed the methylation status of the 5′ upstream region of the miR-145 gene. In prostate cancer cell lines, PC3, LNCaP and Du145, there are 10 CpG sites in the 5′ upstream region of miR-145. In the first 100 bases proximal to the start site, all the CpG sites were found to be completely methylated in these cell lines (Figure 2). In PC3 cells the CpG sites in the second 100 bases were also completely methylated, whereas in the other two the percentage of methylation of these CpG sites decreased to 40% in LNCaP cells. We also checked the methylation levels in prostate carcinoma tissue samples. Out of the 10 samples checked, all had high levels of methylation (>70–80%, data not shown). To see whether the low level of miR-145 in prostate cancer cell lines is because of methylation, we treated PC3, LNCaP and Du145 cells with the demethylating agent 5-aza deoxycytidine. After extracting the total miRNA from these treated cell lines, real-time PCR showed an increase in expression of miR-145 levels of approximately 148, 18- and 64-fold in Du145, LNCaP and PC3 cells, respectively, when compared with untreated cells (Figure 1B). This suggests that methylation of the 5′ upstream regions of the miR-145 gene is one of the factors responsible for its low expression in prostate cancer cells.

### Effect of genistein, 5-aza and TSA on miR-145 expression

**Figure 3:**
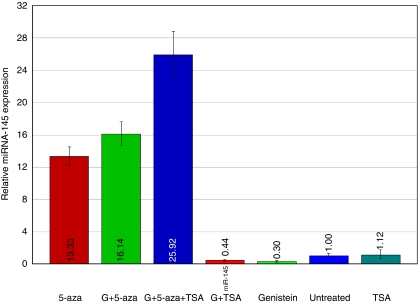
Effect of genistein, 5-aza and TSA on miR-145 expression in PC3 cells. Genistein in combination with 5-aza and TSA significantly increased miR-145 levels.

Owing to the toxicity of the individual treatment agents (genistein, 5-aza and TSA) at high concentrations, all the compounds were used at a comparatively low dose. PC3 cells were treated with genistein (25 *μ*M), 5-aza (5 *μ*M) and TSA (100 ng  *μ*l^–1^), individually and in combination for 96 h. Genistein and 5-aza were administered for the total time period, whereas TSA was administered for the last 24 h. Subsequently, total miRNA was extracted and the level of miR-145 was analysed by real-time PCR. The expression level of miR-145 increased significantly in 5-aza-treated cells (Figure 3) and the highest levels were observed in combination with genistein, 5-aza and TSA (Figure 3).

### Effect of overexpression of miR-145 in PC3 cells

**Figure 4:**
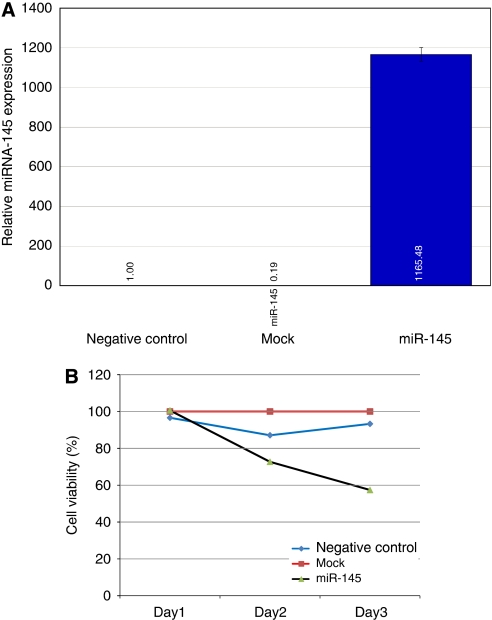
Overexpression of miR-145 in PC3 cells. (**A**) Increase in the level of miR-145 after transfection in PC3 cells. (**B**) Decrease in cell viability in a time-dependent manner in miR-145-transfected cells compared with the negative control and mock samples.

To see the effect of overexpression of miR-145 on prostate cell lines, pre-miR-145, a precursor of miR-145, was transfected into PC3 cells. Real-time PCR analysis of miR-145 expression 72 h after transfection showed expression of miR-145 at >1000-fold over control (Figure 4A). We monitored cellular proliferation after miR-145 upregulation in PC3 cells and found that cell viability significantly decreased in a time-dependent manner in miR-145-transfected cells compared with the negative control and mock samples (Figure 4B). The data were normalised to the mock sample. This suggests that the attenuation of miR-145 expression has an antiproliferative effect on PC3 cells.

**Figure 5:**
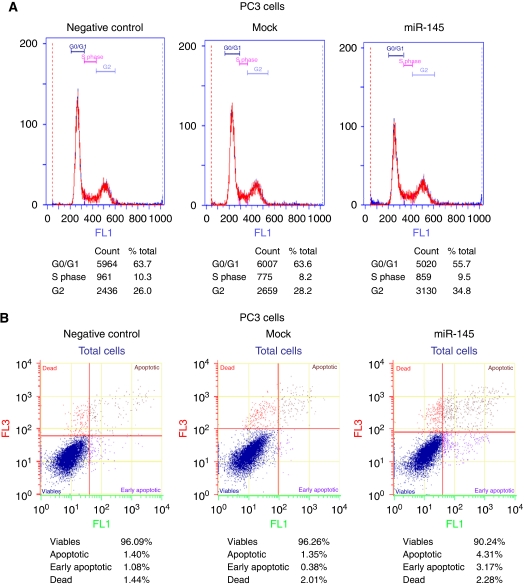

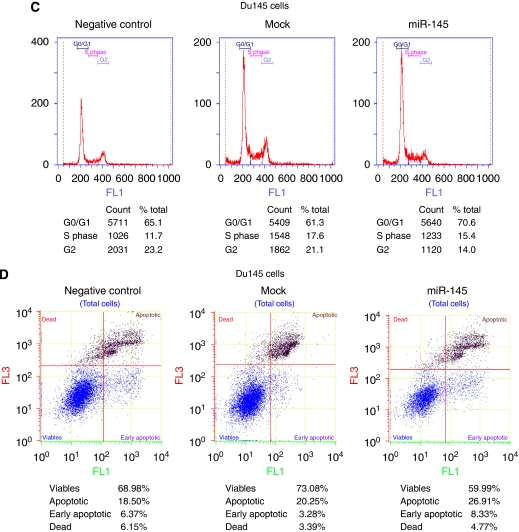
Flow cytometry analysis of miR-145-transfected PC3 cells. (**A**) Cell cycle analyses showed a decrease in the G_0_–G_1_ stage with a concomitant increase in cells in the G_2_–M phase in those cells transfected with a precursor of miR-145 when compared with both mock and negative control. (**B**) miR-145 upregulation increased apoptosis (5%) in the total number of PC3 cells undergoing apoptosis (apoptotic and early apoptotic) in miR-145-transfected PC3 cells when compared with both negative control and mock. (**C**) Cell cycle analysis of miR-145-transfected Du145 cells showed an increase of ∼9% in the cells in G_0_–G_1_ stage when compared with mock, with a concomitant decrease of ∼7% of cells in the G_2_–M phase. (**D**) There was a significant increase (∼11%) in the total number of Du145 cells undergoing apoptosis (apoptotic and early apoptotic) in the miR-145-transfected Du145 cells when compared with mock.

The effects of miR-145 overexpression on the cell cycle and apoptosis were analysed using flow cytometry. Cell cycle analyses showed a decrease in the G_0_–G_1_ stage with a concomitant increase in cells in G_2_–M phase in case of the cells transfected with a precursor of miR-145 when compared with both mock and the negative control (Figure 5A). The effect of miR-145 upregulation on apoptosis showed a 5% increase in the total number of cells undergoing apoptosis (apoptotic and early apoptotic) in miR-145-transfected cells when compared with both negative control and mock (Figure 5B). We also checked the effect of upregulation of miR-145 in prostate cancer cell lines, LNCaP and Du145. No significant effect was observed in the case of LNCaP cells in both cell cycle and apoptosis. Although, in the case of Du145 cell cycle analysis showed an increase of ∼9% in the cells in G_0_–G_1_ stage, in miR-145-transfected cells, when compared with mock, it showed a concomitant decrease of ∼7% in cells in G_2_–M phase (Figure 5C). Apoptosis showed a significant increase (∼11%) in the total number of cells undergoing apoptosis (apoptotic and early apoptotic) in the miR-145-transfected cells when compared with mock (Figure 5D).

### mRNA microarray of miR-145-overexpressing PC3 cells

Micro RNAs are known to have numerous cellular targets. Therefore, we analysed the changes occurring as a result of miR-145 overexpression in PC3 cells using mRNA microarray (data not shown). These results showed the upregulation of a number of pro-apoptotic genes such as *TNFSF10* and *IL-24* (Pitti *et al*, 1996; Sauane *et al*, 2004), and cyclin binding ubiquitin ligases such as HERC5 (Hochrainer *et al*, 2008). There was also downregulation of antiapoptotic genes such as *BIRC5* (Tamm *et al*, 1998), also known as survivin and of the prometastatic gene *FSCN1* (Hashimoto *et al*, 2009).

### TNFSF10 is increased after miR-145 transfection in PC3

**Figure 6:**
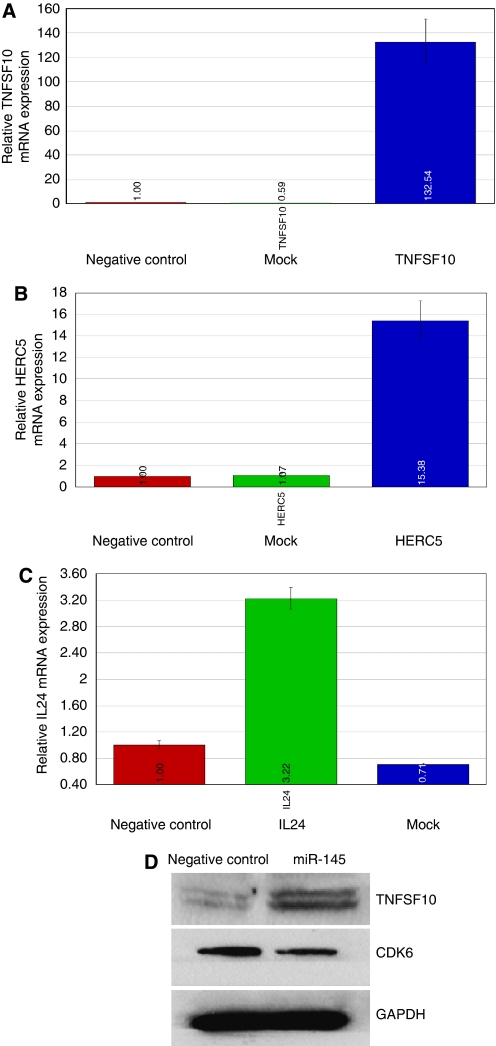
Effect of miR-145 overexpression on apoptosis/cell cycle genes in PC3 cells. Increased expression of (**A**) TNFSF10, (**B**) HERC5 and (**C**) IL24 in miR-145-transfected PC3 cells when compared with mock and the negative control. (**D**) Western analysis of upregulated and downregulated genes in miR-145-transfected PC3 cells. Upregulation of TNFSF10 protein expression occurred concomitantly with downregulation of cdk6 in PC3 cells transfected with miR-145 precursor.

The results of the microarray were validated by real-time PCR. The mRNA expression level of TNFSF10, HERC5 and IL-24 was found to be upregulated by approximately 132-, 16- and 3.5-fold, respectively, by the transfection of miR-145 in PC3 cells (Figure 6A–C). TNFSF10, a member of the family of TRAIL (TNF-related apoptosis-inducing ligands) molecules (Pitti *et al*, 1996) had the maximum increase in mRNA levels by real-time PCR. Its expression was further substantiated by checking the level of protein expression of TNFSF10 by western blotting in the transfected PC3 cells. Protein expression of TNFSF10 was found to be substantially upregulated for the miR-145 sample when compared with the negative control (Figure 6D). Moreover, we also checked the protein expression of different genes that are involved in regulating apoptosis and cell cycle. Among these were survivin, Bcl-2, cdc7, cdk6 and PUMA. Out of these, only cdk6 showed a significant decrease in its expression in the miR-145-transfected sample when compared with the negative control (Figure 6D).

## Discussion

Prostate cancer accounts for approximately 29% of cancer incidence and is second only to lung cancer in male cancer deaths in the United States (Jemal *et al*, 2007). Treatments available for this disease include hormone therapy, radical prostatectomy or irradiation. Generally, a subset of prostate cancer patients is prone to disease relapse and metastasis, which frequently develops even after surgery and may cause death. Therefore, a number of alternative ways for controlling prostate cancer have emerged, including chemoprevention and delaying or reversing carcinogenesis by pharmacologic intervention with naturally occurring or synthetic agents (Sporn and Suh, 2002; Tsao *et al*, 2004). Tumourigenesis is a multistep process that results from the accumulation and interplay of genetic and epigenetic changes. Increased DNA methylation of CpG islands in the promoter region of genes is well established as a common epigenetic mechanism for the silencing of tumour suppressor genes (TSGs) in cancer cells (Jones and Laird, 1999). Epigenetic silencing of a gene can be reversed by drugs such as 5-aza-2′-deoxycytidine (5Aza-C) that forms a covalent complex with the active site of methyltransferase resulting in generalised demethylation. Unfortunately, the applicability of this commonly used drug is hampered by its high toxicity and instability in physiological solutions (Bender *et al*, 1998). Histone deacetylases (HDACs) are frequently overexpressed in a broad range of cancer types, in which they alter cellular epigenetic programming to promote cell proliferation and survival (Marks *et al*, 2001). HDAC inhibitors are presently approved for treatment of advanced primary cutaneous T-cell lymphoma (Mann *et al*, 2007), and have been shown to be effective in animal models of prostate cancer (Wedel *et al*, 2008). In a previous study, it has been shown that treatment of prostate cancer cells with the HDAC inhibitor TSA induces cell cycle arrest but not apoptosis (Roy *et al*, 2005).

Epidemiologic evidence suggests that intake of a soy-rich diet may have a protective effect against prostate cancer (Hebert *et al*, 1998; Jacobsen *et al*, 1998). Genistein, one of the principal soy isoflavones, shows a wide array of chemopreventive actions. The anticancer effects of genistein have been ascribed to several signalling pathways and mechanisms that lead to cell cycle arrest, apoptosis, invasion, metastasis and angiogenesis, the attributes that could potentially prevent tumour initiation and progression (Magee and Rowland, 2004; Bektic *et al*, 2005). Genistein (4′,5,7-trihydroxyflavone) is a naturally occurring isoflavenoid that is abundant in soy products and has been identified as an inhibitor of protein tyrosine kinases and thus it has a key role in cell growth and apoptosis (Hunter, 1987; Ullrich and Schlessinger, 1990). It has been reported to have estrogenic properties and antineoplastic activity in multiple tumour types (Zava and Duwe, 1997). It was also found to have epigenetic effects in the mouse prostate (Day *et al*, 2002). The above findings prompted us to examine its effect on the expression of miRNAs. Furthermore, the role of 5-aza and TSA in the reversal of epigenetic silencing of genes prompted us to use them in combination with genistein.

Micro RNAs have important roles in many biological processes, including cell growth, apoptosis, haematopoietic lineage differentiation and gene regulation. They are also involved in a wide variety of human diseases such as cancer, vascular disease, immune disease and infections. More than 50% of human miRNA genes are thought to be located in cancer-associated regions or at fragile sites of chromosomes, which are hot spots for gene deletion, amplification and mutations. Many genomic profiling studies have indicated a general downregulation of miRNAs in various tumours, including pancreatic cancer, breast cancer, prostate cancer, liver cancer, colon cancer and ovarian cancer, suggesting a more negative regulation of cancer growth by miRNAs (Gramantieri *et al*, 2007; Hurteau *et al*, 2007; Lee *et al*, 2007; Shi *et al*, 2007; Barbarotto *et al*, 2008; Felicetti *et al*, 2008; Mitomo *et al*, 2008; Schetter *et al*, 2008; Yang *et al*, 2008).

The consistency with which miR-145 was found to be downregulated in both prostate cell lines and carcinoma samples prompted us to examine the role it has in prostate cancer. It is evident from our results that loss of miR-145 might be important in the development and progression of prostate cancer, as it's overexpression resulted in an increase in the total number of cells undergoing apoptosis. This led us to hypothesise that miR-145 might be affecting apoptosis. mRNA microarray of the overexpressed miR-145 in PC3 cells confirmed our hypothesis when we observed the upregulation of pro-apoptotic genes such as *TNFSF10* and *IL-24*. Real-time PCR of TNFSF10 mRNA showed upregulation of its expression (132-fold), which was confirmed by western blotting. TNFSF10, also known as TRAIL or Apo-2 ligand (Pitti *et al*, 1996), is a member of the TNF superfamily, and since its discovery it has been used as an antitumour protein because of its ability to induce apoptosis in a variety of human cancer cell lines while leaving normal cells unaffected (Walczak *et al*, 1999). Moreover, studies performed on TRAIL knockout mice show that TNFSF10 has an important role in normal tumour immunosurveillance as TNFSF10 knockout mice support tumour growth at a higher rate when compared with normal mice and are more susceptible to tumour metastasis (Cretney *et al*, 2002). Cells undergoing TRAIL-induced death show many of the hallmarks of apoptosis, including DNA fragmentation, expression of pro-phagocytic signals on the cell membrane and cleavage of multiple intracellular proteins by caspases (Griffith *et al*, 1998).

In summary, our results suggest methylation of the promoter region of miR-145 in prostate cancer cell lines and prostate cancer tissue samples as a major reason for its low expression. This is the first report on the mechanism of inactivation of miR-145 through DNA methylation as well as the functional significance of miR-145 in prostate cancer. As one of the genes significantly upregulated by miR-145 overexpression is the pro-apoptotic *TNFSF10* gene, modulation of miR-145 may be an important therapeutic approach for the management of prostate cancer.
