# The functional significance of microRNA-145 in prostate cancer

**DOI:** 10.1038/bjc.2011.71

**Published:** 2011-03-01

**Authors:** M S Zaman, Y Chen, G Deng, V Shahryari, S O Suh, S Saini, S Majid, J Liu, G Khatri, Y Tanaka, R Dahiya

**Correction to**: *British Journal of Cancer* (2010) **103**, 256–264. doi: 10.1038/sj.bjc.6605742

Upon publication of this paper in mid 2010, the authors noted the omission of a grant number in the Acknowledgments section of their paper, which they would now like to correct. They hereby acknowledge the help of the NIH grant number RO1CA138642.

The authors would like to apologise for any inconvenience caused.

